# Patient-Reported Outcomes-Guided Adaptive Radiation Therapy for Head and Neck Cancer

**DOI:** 10.3389/fonc.2021.759724

**Published:** 2021-10-19

**Authors:** Sarah Weppler, Harvey Quon, Colleen Schinkel, Adam Yarschenko, Lisa Barbera, Nabhya Harjai, Wendy Smith

**Affiliations:** ^1^ Department of Physics and Astronomy, University of Calgary, Calgary, AB, Canada; ^2^ Department of Medical Physics, Tom Baker Cancer Centre, Calgary, AB, Canada; ^3^ Department of Radiation Oncology, Tom Baker Cancer Centre, Calgary, AB, Canada; ^4^ Department of Oncology, University of Calgary, Calgary, AB, Canada; ^5^ Department of Mechanical Engineering, University of Calgary, Calgary, AB, Canada; ^6^ Cumming School of Medicine, University of Calgary, Calgary, AB, Canada

**Keywords:** patient-reported outcomes, adaptive radiation therapy, head and neck cancer, dysphagia, xerostomia

## Abstract

**Purpose:**

To identify which patient-reported outcomes (PROs) may be most improved through adaptive radiation therapy (ART) with the goal of reducing toxicity incidence among head and neck cancer patients.

**Methods:**

One hundred fifty-five head and neck cancer patients receiving radical VMAT (chemo)radiotherapy (66-70 Gy in 30-35 fractions) completed the MD Anderson Symptom Inventory, MD Anderson Dysphagia Inventory (MDADI), and Xerostomia Questionnaire while attending routine follow-up clinics between June-October 2019. Hierarchical clustering characterized symptom endorsement. Conventional statistical approaches indicated associations between dose and commonly reported symptoms. These associations, and the potential benefit of interfractional dose corrections, were further explored *via* logistic regression.

**Results:**

Radiotherapy-related symptoms were commonly reported (dry mouth, difficulty swallowing/chewing). Clustering identified three patient subgroups reporting: none/mild symptoms for most items (60.6% of patients); moderate/severe symptoms affecting some aspects of general well-being (32.9%); and moderate/severe symptom reporting for most items (6.5%). Clusters of PRO items broadly consisted of acute toxicities, general well-being, and head and neck-specific symptoms (xerostomia, dysphagia). Dose-PRO relationships were strongest between delivered pharyngeal constrictor Dmean and patient-reported dysphagia, with MDADI composite scores (mean ± SD) of 25.7 ± 18.9 for patients with Dmean <50 Gy *vs*. 32.4 ± 17.1 with Dmean ≥50 Gy. Based on logistic regression models, during-treatment dose corrections back to planned values may confer ≥5% decrease in the absolute risk of self-reported physical dysphagia symptoms ≥1 year post-treatment in 1.2% of patients, with a ≥5% decrease in relative risk in 23.3% of patients.

**Conclusions:**

Patient-reported dysphagia symptoms are strongly associated with delivered dose to the pharyngeal constrictor. Dysphagia-focused ART may provide the greatest toxicity benefit to head and neck cancer patients, and represent a potential new direction for ART, given that the existing ART literature has focused almost exclusively on xerostomia reduction.

## 1 Introduction

Standard-of-care (chemo)radiotherapy is associated with a high toxicity burden for many locally-advanced head and neck cancer patients. Physician assessments suggest that ≥30% of patients will experience grade 2 or worse radiation-associated dysphagia (1) with ≥35% experiencing grade 2 or worse xerostomia (2). Volumetric modulated arc therapy (VMAT) provides dose-sculpting capabilities to reduce incidental radiation doses to healthy tissues (2); however, decreases in tumor volume (3), weight loss (4), and other inter-fractional anatomical changes common among head and neck cancer patients may reduce treatment precision and increase toxicity (5, 6). Reduction of treatment-related side effects is increasingly important given the rise of HPV-related disease (7), as well as younger age and improved prognosis of these patients (8).

Adaptive radiation therapy (ART) adapts a patient’s radiotherapy plan in response to inter-fractional anatomical changes to maintain target coverage and healthy tissue dose sparing objectives during the 6-7 week treatment course. ART may improve the therapeutic ratio of radiotherapy (3) and reduce treatment-related toxicities (5), but is resource intensive (9). Effective patient selection is therefore essential for ensuring that ART is feasible in a routine clinical setting. However, many open questions remain regarding patient selection: even in a broad sense, it is unclear which toxicity ART may most reduce.

When considering toxicity-reduction strategies, such as ART, patient-report outcomes (PRO) provide valuable insight into symptom burden. Physician assessments are essential for patient care but may underreport symptom severity relative to patient reporting (10). PROs help to fill the gap by providing the patient’s perspective of the impact of symptoms and toxicity on daily patient life (11, 12). Examples of PRO instruments include the MD Anderson Symptom Inventory for Head and Neck Cancer (MDASI-HN) (13, 14), the MD Anderson Dysphagia Inventory (MDADI) (15) and the Xerostomia Questionnaire (XQ) (16). These instruments are widely used and score highly in reliability, validity, and responsiveness to changes over time (13–17).

In this study, we compare planned doses, delivered doses, and PROs (MDASI-HN, MDADI, and XQ) to identify which patient-reported side-effects may be most improved by ART, and to estimate the associated toxicity benefit. It is our hope that these results will provide further structure to the development of ART workflows and effective patient-selection criteria.

## 2 Materials and Methods

### 2.1 Patient Inclusion Criteria

Patients attending routine radiotherapy follow-up appointments between June and October 2019 were approached to complete a one-time paper-based PRO questionnaire in clinic. The questionnaire consisted of the MDASI-HN, MDADI, and XQ. Patients included in this study received treatment with radical VMAT (chemo)radiotherapy (66-70 Gy in 30-35 fractions). Patients were excluded if they were treated with a dose prescription less than 66 Gy, did not receive CBCT imaging, or had a confirmed local-regional recurrence prior to survey completion. This study was approved by our institutional research ethics board (HREBA.CC-19-0119).

### 2.2 Exposure Definition – Planned and Delivered Dose

Planned organ-at-risk (OAR) dose parameter values were extracted from the patient’s treatment plan. OAR planning objectives adhered to QUANTEC and other consensus recommendations and included: brainstem D0.03cc ≤ 54 Gy (18); spinal cord D0.03cc ≤ 45 Gy (19); ipsilateral and contralateral parotid gland Dmean ≤ 26 Gy (20, 21); and pharyngeal constrictor Dmean ≤ 50 Gy (22). Treatments were planned using the Eclipse Treatment Planning System, Versions 11 and 13 (Varian Medical Systems, Palo Alta, CA). Institutional image-guided radiation therapy utilized daily kV-orthogonal imaging and weekly kV-cone beam CT (CBCT) imaging (23).

Previously validated deformable image registration workflows allowed us to estimate delivered OAR doses (23). For each patient, we deformed a copy of the planning CT to reproduce the anatomical changes present in the last-acquired on-unit CBCT. We propagated contours through the corresponding deformation vector mapping, re-applied the patient’s treatment plan, and recalculated dose in the treatment planning system. These doses served as a surrogate for total delivered dose. Assuming that patient anatomy was consistent with the final CBCT for all treatment fractions provided conservative estimates for the associations between dose and PROs. Quality assurance of this process assessed a representative set of cases (24), and ensured the propagated structures were geometrically (25) and dosimetrically (26) consistent with physician contours (23).

### 2.3 Outcome Definition – Patient-Reported Outcome Instruments

The MDASI-HN consists of 28 questions assessing core symptoms (13 items), head and neck-specific symptoms (9 items), and symptom interference on daily life (6 items) (13, 14). Each item is ranked from 0 to 10 with symptom burden interpreted as: none (item rating of 0); mild (1 to 4); moderate (5 to 6); or severe (7 to 10) (13). Summary symptom burden is defined by the maximum rating of any item within each subgroup: none (all items rated 0); mild (all items rated <5 with at least one item rated ≥1); moderate (all items rated <7 with at least one item rate ≥5); severe (at least one item rated ≥7) (27–29).

The MDADI contains 20 questions assessing physical swallowing ability (8 items), functional impact of swallowing dysfunction (5 items), emotional impact (6 items), and the general influence of swallowing ability on daily life (1 item) (15). Ratings for physical, functional, and emotional items are summed to produce the composite score (15). For this study, 5-point Likert-responses were normalized to 100 with higher scores indicating more severe symptoms. This provided greater comparability with the MDASI-HN and XQ scoring systems. With this conversion, MDADI scores are interpreted as: minimal (summary score of 0 to 19), mild (20 to 39), moderate (40 to 59), severe (60 to 79), and profound (80 to 100) (30, 31). Differences in MDADI scores ≥10 points are considered clinically relevant (32). References to MDADI moderate/severe scores below also include scores classified as “profound”.

The XQ is an 8-item assessment of xerostomia symptoms while eating (4 items) and while not eating (4 items). Item scores are totaled and normalized to 100 (16). Symptom burden according to XQ responses was interpreted as: none/mild for scores <50 and moderate/severe for scores ≥50).

### 2.4 Covariates – Clinical Patient Characteristics

Data for this study consisted of basic demographic and tumor factors abstracted from the patient’s medical record. These included patient: age; gender; BMI; ECOG performance status; Charlson Comorbidity Index; tobacco/alcohol use; tumor site and stage; HPV status; and chemotherapy agent.

### 2.5 Data Clustering, Statistical Analysis, and Logistic Regression Modelling

#### 2.5.1 Characterization of Patient-Reported Outcomes

Using Mann-Whitney U tests and Fisher’s exact tests, we examined potential associations between clinical characteristics and PRO item and summary scores. Benjamini-Hochberg multiple testing corrections were applied with a false discovery rate of 5% (33).

Hierarchical clustering tested for similarities in symptom reporting among PRO items and summary scores, as well as symptom burden among patients. This technique progressively groups items considered most similar, as represented in tree-like “dendrograms” (34). Similarities in PRO results were used to: characterize PRO reporting; verify dose-PRO associations among related PRO items; and identify similarities in patient symptoms to examine the effect of covariates.

#### 2.5.2 Associations Between Planned Dose, Delivered Dose and Patient-Reported Outcomes

We stratified patients according to whether their OAR dose met *vs*. exceeded planning objective criteria. Differences in PRO scores between these groups were compared using Mann-Whitney U tests. Odds ratios indicated whether patients with OAR dose exceeding planning objectives had a greater likelihood of reporting moderate/severe symptoms, with significance from Fisher’s exact tests. Tests were performed for both planned dose and delivered dose. For parotid gland doses, we compared the dose of the spared gland (i.e., the lesser of ipsilateral and contralateral gland Dmean values) with PRO results.

As moderate/severe symptoms persisting ≥1 year after treatment are more likely to be permanent (35, 36), we further assessed differences in patients completing the PRO questionnaire <1 year *vs*. ≥1 year post-treatment.

#### 2.5.3 Estimating the Benefit of Adaptive Replanning

When delivered OAR doses were found to be strongly associated with PRO scores, we estimated the potential benefit of ART on patient-reported symptom severity. Systematic dose increases considered potentially correctable by replanning (dose “violations”) were calculated relative to planning objectives and planned values, as relevant to clinical practice and QUANTEC guidelines. Additional tolerances accounted for random errors in estimated delivered doses to produce conservative estimates of ART benefit. For our given workflow, calculated increases in parotid gland dose exceeding 2.2 Gy, and pharyngeal constrictor dose exceeding 0.75 Gy are likely to result from systematic changes in patient anatomy, as compared to daily setup uncertainties or deformable image registration error (23). For patients with planned doses meeting planning objectives,

Violation =delivered dose – planning objective – random error tolerance (1) For example, a patient with planned pharyngeal constrictor dose of 49.0 Gy and estimated delivered dose of 52.0 Gy would have a 1.25 Gy violation. For patients with planned doses exceeding planning objectives,

Violation = delivered dose – planned dose – random error tolerance (2) Therefore, a patient with planned pharyngeal constrictor dose of 54.0 Gy and estimated delivered dose of 57.0 Gy would have a 2.25 Gy violation. Positive violation values indicate the amount of dose sparing achievable with adaptive dose corrections; patients with positive violations likely have increased risk of treatment-related side effects relative to that estimated at planning. Negative values indicate that: only minor dose increases occurred during treatment as a result of random effects; delivered structure dose corresponded to a relatively low-risk of toxicity (i.e., delivered doses met the treatment planning objective); or that dose and corresponding toxicity risk decreased during treatment.

Logistic regression was used to model dose violations versus risk of moderate/severe symptom reporting. For each patient, the risk of moderate/severe symptom reporting was estimated for raw delivered doses and doses corrected back to planned values; corresponding differences in risk indicated the potential benefit, if any, of ART on patient-reported symptom severity.

All analyses were performed using R Version 3.6.0 (The R Foundation for Statistical Computing, Vienna, Austria). All statistical tests required p ≤ 0.05 for significance.

## 3 Results

### 3.1 Cohort Characteristics and Characterization of Patient-Reported Outcomes

**Figure 1:**
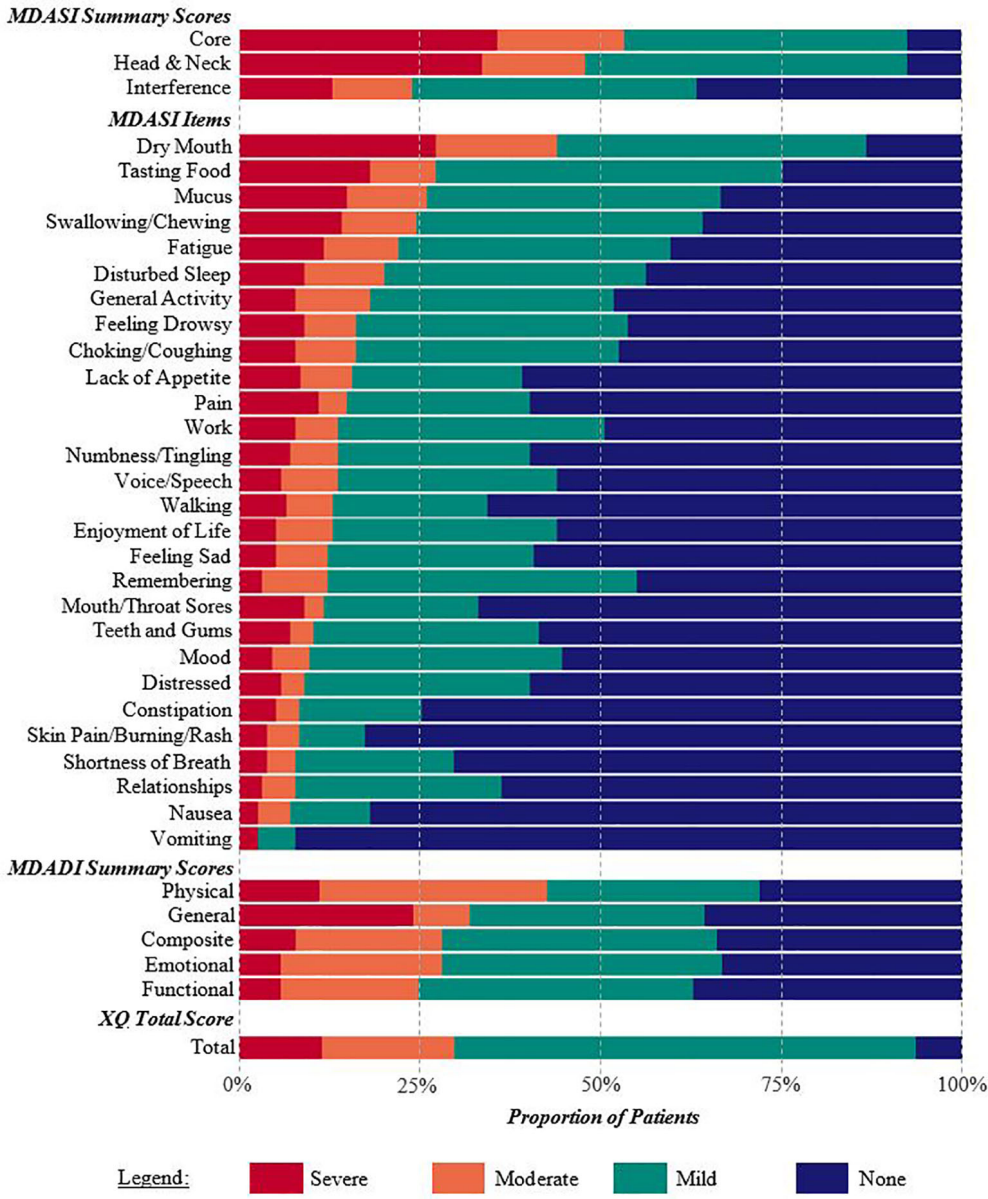
Percentage of patients reporting none, mild, moderate, or severe symptoms on the MDASI-HN, MDADI, and XQ. Summary scores and individual items are listed according to the proportion of patients with moderate or severe symptoms. Xerostomia and dysphagia-related symptoms were commonly reported.

225 patients completed the PRO questionnaires in clinic. After applying the inclusion/exclusion criteria, the final study cohort consisted of 155 patients. **Table 1** provides cohort demographics and characteristics. MDASI-HN, MDADI, and XQ results are summarized in **Figure 1**. 60 patients completed the PRO questionnaire within their first year after treatment (median = 7 months, range = 2-11 months), with the remaining 95 patients completing the questionnaire ≥1 year post-treatment (28 months, 12-74 months).

**Table 1:** Cohort demographic and clinical characteristics.

Parameter	Full Cohort (n = 155)
Age in years, mean (±SD)	57.4 (10.9)
Gender, number (%)	
Male	131 (84.5%)
Female	24 (15.5%)
Initial BMI, mean (±SD)	28.1 (5.6)
ECOG, median (range)	1 (1-3)
Charlson Comorbidity Index, median (range)	4 (2-8)
Alcohol use, number (%)	
Never	36 (23.2%)
Former	12 (7.7%)
Current – Light (males 0-15 drinks/week, females 0-10 drinks/week)	83 (53.6%)
Current – Heavy (males >15 drinks/week, females >10 drinks/week)	24 (15.5%)
Tobacco use, number (%)	
Never	63 (40.7%)
Cumulative – Light (0-20 pack-years)	43 (27.7%)
Cumulative – Heavy (>20 pack-years)	49 (31.6%)
Primary tumor location, number (%)	
Larynx	7 (4.5%)
Hypopharynx	3 (1.9%)
Oral Cavity	3 (1.9%)
Oropharynx	98 (63.3%)
Nasal Cavity	7 (4.5%)
Nasopharynx	26 (16.8%)
Unknown	11 (7.1%)
T stage, number (%)	
T0 – T2	71 (45.8%)
T3 – T4	73 (47.1%)
Tx	11 (7.1%)
N stage, number (%)	
N0	23 (14.8%)
N1	34 (21.9%)
N2	83 (53.6%)
N3	14 (9.0%)
NX	1 (0.7%)
p16 status, number (%)	
Negative	21 (13.6%)
Positive	100 (64.5%)
Unknown	34 (21.9%)
Chemotherapy agent, number (%)	
Carboplatin	3 (1.9%)
Cetuximab	13 (8.4%)
Cisplatin (Cisplatinum)	128 (82.6%)
None	11 (7.1%)
Time Since Treatment, median (range)	18 months (2-74 months)

Patients with lower initial BMI or poorer performance status more frequently reported moderate/severe fatigue, sadness, poorer activity, greater interference of symptoms with work, and poorer overall interference with daily life (p < 0.005 for each) on the MDASI-HN. Greater T stage (T3-T4 disease) was significantly associated with higher MDADI composite summary scores (p < 0.005). No statistically significant differences occurred in clinical parameters for other MDASI-HN, MDADI or XQ responses, including HPV status and time since treatment, according to Mann-Whitney U tests and Fisher’s exact tests.

**Figure 2:**
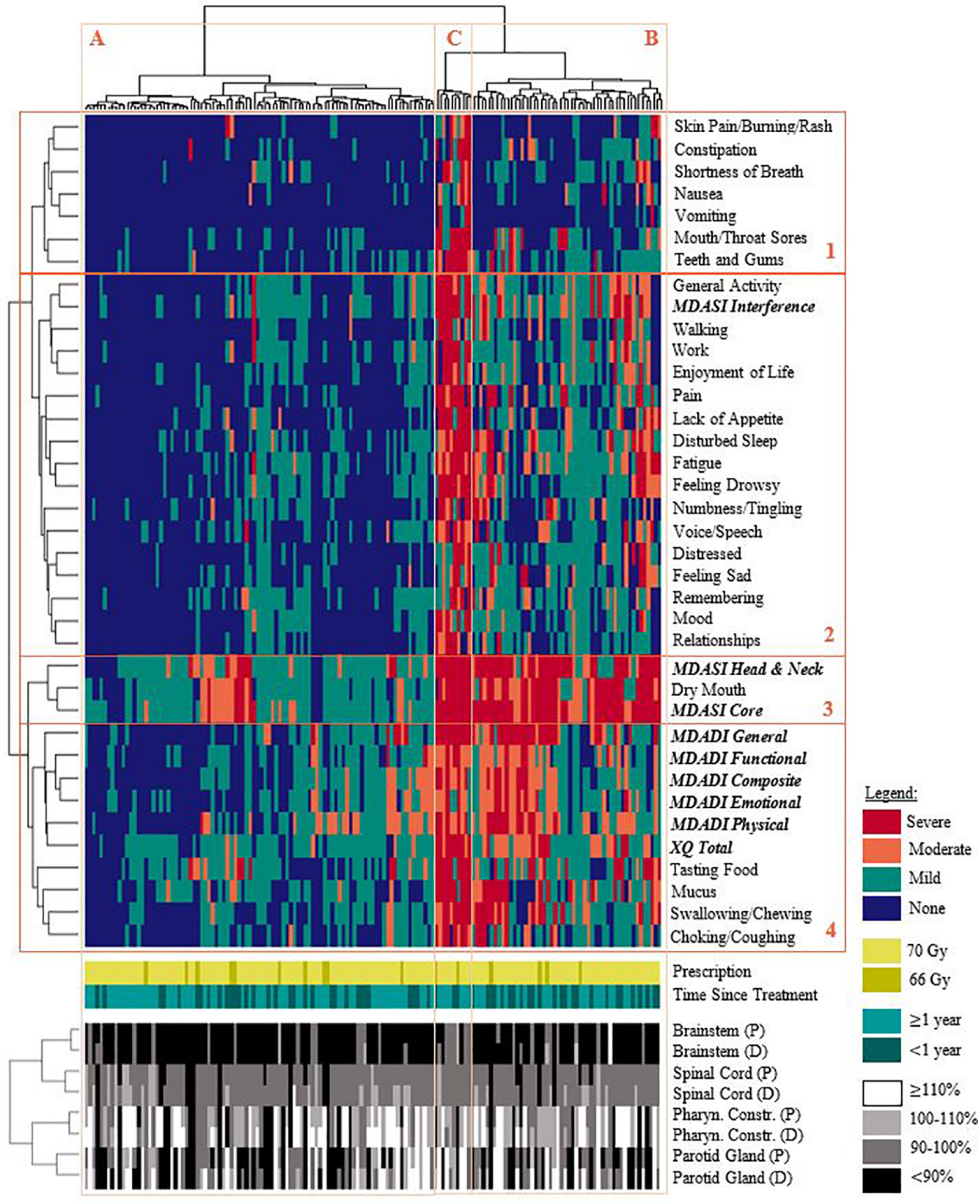
Hierarchical clustering of patient-reported symptoms (none/mild/moderate/severe), prescription dose, time since completing treatment, and OAR dose. Each row (groups 1-4) represents a specific symptom or summary score and are clustered as: 1.) acute toxicities, 2.) general wellbeing, 3.) xerostomia-related summary scores, 4.) xerostomia and dysphagia-related symptoms. Each column represents a patient in the cohort; patients generally reported: **(A)** none/mild symptoms for most/all items, **(B)** moderate/severe symptom burden affecting some aspects of general wellbeing, **(C)** moderate/severe symptom reporting for most/all items. Delivered dose generally exceeded planned dose. Note: healthy tissue doses are expressed as relative percentages of the planning objective. (P): planned dose. (D): delivered dose.

Results of the hierarchical clustering are shown in **Figure 2**. PRO items were grouped according to: acute side-effects, general wellbeing, and xerostomia/dysphagia-related toxicities, with the latter combining various MDASI-HN, MDADI, and XQ items. The MDASI-HN dry mouth item strongly contributed to the MDASI-HN core and head and neck summary scores. Clustering indicated three general symptom profiles: none/mild symptoms for the majority of items (Cluster A, 60.6% of patients); moderate/severe symptoms affecting some aspects of general wellbeing (Cluster B, 32.9%); and moderate/severe symptom reporting for most items (Cluster C, 6.5%). Patients in cluster C were younger on average (49.8 years, p = 0.04), while patients in cluster A had a greater proportion of non-smokers (46.8%, p = 0.03). 6 of the 10 patients in cluster C, reporting moderate/severe symptoms for most items, had nasopharyngeal disease and greater planned and delivered brainstem dose although this was not found to be statistically significant. No other statistically significant differences persisted among the clinical, geometric, or dosimetric characteristics between clusters after multiple testing corrections.

### 3.2 Associations Between Planned Dose, Delivered Dose and Patient-Reported Outcomes

**Table 2** summarizes the associations between OAR dose and PRO responses. Stratifying patients based on whether their planned pharyngeal constrictor doses met *vs*. exceeded the planning objective revealed statistically significant differences in MDADI composite, physical, and functional summary scores. These differences persisted for delivered pharyngeal constrictor dose, with additional statistical significance in emotional summary scores. Independently calculated odds ratios were statistically significant for MDADI physical and emotional scores with respect to both planned and delivered doses. Odds ratios associated with delivered doses exceeded those for planned doses, suggesting that delivered dose may be more strongly associated with these PRO summary scores. For MDADI composite scores, odds ratios had marginal significance for both planned dose (OR = 2.02, p = 0.09) and delivered dose (OR = 2.26, p = 0.06).

**Table 2:** Comparison of patient-reported symptom scores and dose, reported as mean (SD) for patients with dose meeting *vs*. exceeding planning objectives.

Toxicity/OAR (Obj.)	Relevant PROMs	Planned Dose		Delivered Dose	
		(<Obj./≥ Obj.)	OR (95% CI)	(<Obj./≥ Obj.)	OR (95% CI)
Xerostomia/ Parotid Glands (Dmean ≤ 26 Gy)	% Patients (n = 150)	67.7% (100)/32.3% (50)	N/A	55.5% (81)/44.5% (69)	N/A
	Average Dose	20.1 Gy/30.8 Gy	N/A	19.3 Gy/31.9 Gy	N/A
	*MDASI Summary Scores and Relevant Items*				
	Core	5.1 (3.2)/4.9 (3.2)	0.73 (0.38-1.39)	5.3 (3.3)/4.8 (3.0)	0.93 (0.49-1.77)
	• Dry Mouth	4.3 (3.3)/4.4 (3.2)	0.75 (0.40-1.44)	4.5 (3.4)/4.1 (3.0)	0.92 (0.48-1.76)
	Head & Neck	4.7 (3.2)/5.0 (3.3)	0.72 (0.38-1.37)	5.0 (3.2)/4.6 (3.2)	0.77 (0.41-1.47)
	• Swallowing/Chewing	2.6 (3.0)/2.9 (3.2)	1.00 (0.48-2.10)	2.6 (3.1)/2.7 (3.0)	1.04 (0.49-2.19)
	• Taste	3.1 (3.1)/3.3 (2.8)	0.88 (0.43-1.81)	3.2 (3.2)/3.0 (2.7)	0.89 (0.43-1.83)
	• Mucus	2.7 (2.9)/2.7 (3.5)	1.00 (0.48-2.08)	2.9 (3.0)/2.5 (3.2)	1.03 (0.49-2.14)
	Interference	2.7 (2.7)/2.4 (3.0)	0.65 (0.30-1.37)	2.7 (2.9)/2.4 (2.8)	0.78 (0.37-1.64)
	*XQ Total Score*	32.3 (23.4)/37.2 (26.9)	1.66 (0.80-3.46)	32.8 (24.0)/35.1 (25.4)	1.70 (0.81-3.57)
Dysphagia/Pharyngeal Constrictor (Dmean ≤ 50 Gy)	% Patients (n = 142)	46.5% (59)/53.5% (83)	N/A	42.6% (53)/57.4% (89)	N/A
	Average Dose	44.3 Gy/56.5 Gy	N/A	44.1 Gy/57.1 Gy	N/A
	*MDASI Summary Scores and Relevant Items*				
	Core	5.1 (3.0)/4.8 (3.3)	0.74 (0.38-1.45)	5.0 (2.9)/4.9 (3.3)	0.91 (0.46-1.82)
	Head & Neck	5.1 (3.1)/4.5 (3.2)	0.65 (0.33-1.26)	5.0 (3.1)/4.6 (3.2)	0.73 (0.37-1.44)
	• Swallowing/Chewing	2.6 (3.0)/2.6 (3.0)	1.02 (0.47-2.23)	2.4 (2.9)/2.7 (3.0)	1.33 (0.59-3.01)
	• Choking/Coughing	1.6 (2.4)/1.9 (2.5)	1.03 (0.41-2.60)	1.7 (2.4)/1.8 (2.5)	1.05 (0.41-2.70)
	• Taste	2.9 (3.1)/3.1 (3.0)	0.97 (0.46-2.06)	2.8 (3.1)/3.2 (3.0)	1.03 (0.48-2.22)
	Interference	2.7 (2.8)/2.4 (2.8)	0.59 (0.27-1.29)	2.8 (3.0)/2.3 (2.7)	0.55 (0.25-1.20)
	*MDADI Summary Scores*				
	Composite	**26.4 (18.7)/32.4 (17.2)**	2.02 (0.90-4.50)	**25.7 (18.9)/32.4 (17.1)**	2.26 (0.97-5.25)
	Physical	**30.9 (22.7)/37.4 (20.3)**	**2.41 (1.18-4.91)**	**29.7 (21.7)/37.6 (20.9)**	**2.70 (1.29-5.68)**
	Emotional	25.2 (18.2)/30.6 (18.7)	**2.52 (1.11-5.72)**	**24.6 (18.5)/30.6 (18.4)**	**2.87 (1.20-6.87)**
	Functional	**20.6 (19.4)/26.7 (18.7)**	1.70 (0.73-3.92)	**20.6 (21.4)/26.3 (17.5)**	1.63 (0.69-3.85)
	General	25.9 (30.0)/33.4 (30.8)	1.41 (0.68-2.93)	26.4 (31.1)/32.6 (30.2)	1.25 (0.60-2.64)

Bold entries indicate that mean values are statistically significant (p ≤ 0.05) according to Mann-Whitney tests, and that odds ratios are statistically significant (p ≤ 0.05) according to Fisher’s Exact tests. Obj.: treatment planning dose objective. OR: odds ratio denoting the odds of moderate/severe responses vs. none/mild responses for doses < Obj. vs. ≥ Obj.

Furthermore, patients with doses meeting *vs*. exceeding the pharyngeal constrictor planning objective had significantly different MDADI scores across all summary items when reporting ≥1 year after treatment completion, with respect to both planned and delivered doses; various MDADI summary scores had mean differences exceeding the 10 point threshold for clinical relevance (32). For planned doses, we observed differences in MDADI composite scores of 13.9; similarly, for delivered doses, we observed differences of 10.7. Mean differences exceeding 10 points also occurred for physical scores (16.3 with respect to planned doses; 13.3 for delivered dose) and general scores (19.8; 14.8). This suggests that pharyngeal constrictor dose meaningfully stratifies patient symptom-reporting ≥1 year post-treatment. Estimating odds ratios associated with PRO scores reported ≥1 year post-treatment was limited by the small number of patients reporting moderate/severe symptoms with doses less than the planning objective.

**Figure 3:**
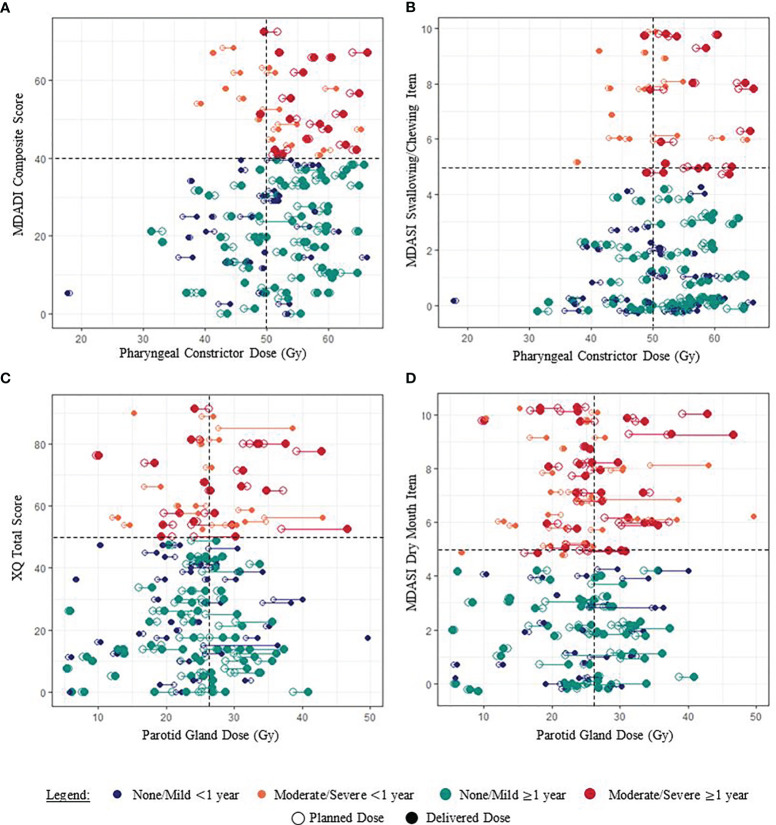
Examples of associations between paired planned and delivered OAR doses and PRO scores for each patient (joined by a horizontal line). **(A, B)** Pharyngeal constrictor doses of patients reporting moderate/severe dysphagia symptoms generally exceeded the planning objective of 50 Gy. **(C, D)** The relationship between parotid gland dose and patient-reported xerostomia symptoms was less clear. Random “jitter” up to ±0.3 has been added to MDASI-HN item scores to better visualize the data.

Among patients with moderate/severe MDADI composite scores, 62.8% had planned pharyngeal constrictor doses exceeding the treatment planning objective, and 67.4% had delivered doses exceeding the objective (**Figure 3A**). In general, delivered doses exceeded planned doses for each patient (**Supplementary Material**). Although not statistically significant, **Figure 3** indicates similar dose and PRO associations for MDASI-HN swallowing/chewing responses, also observed for the MDASI-HN choking/coughing item (not shown), found to be related *via* cluster analysis. Associations appeared strongest among patients reporting ≥1 year after treatment completion.

Patients with minimum parotid gland doses exceeding planning objectives had higher XQ scores, although this was not statistically significant (**Table 2**). No clear associations between parotid gland dose and patient-reported xerostomia symptoms were observed when considering patients in aggregate or according to <1 year *vs*. ≥1 year post-treatment (**Figure 3**).

### 3.3 Estimating the Benefit of Adaptive Replanning

55.6% of patients had non-negative pharyngeal constrictor dose violations. 33.1% of patients had pharyngeal constrictor dose violations exceeding 1 Gy (mean = 1.8 Gy in this cohort subgroup); 8.5% with increases exceeding 2 Gy (mean = 2.8 Gy); and 3.5% with increases exceeding 3 Gy (mean = 3.5 Gy).

**Figure 4:**
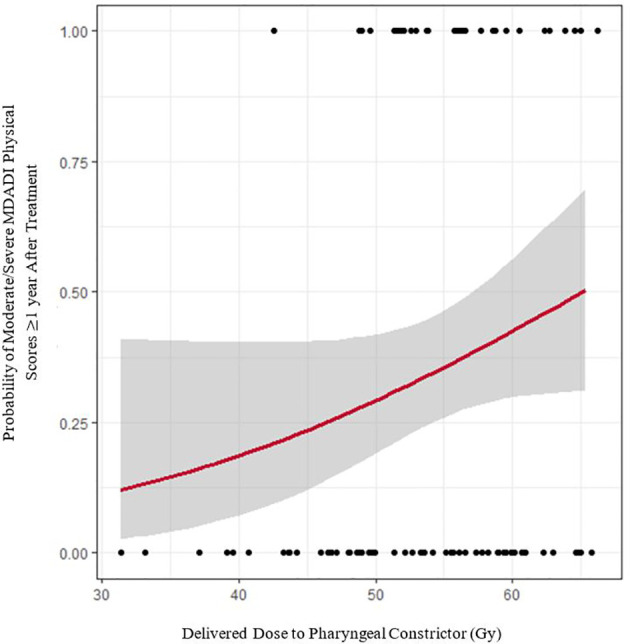
Logistic regression model of delivered pharyngeal constrictor dose versus moderate/severe MDADI physical responses persisting ≥ 1 year after treatment (red line). Grey error bands indicate the 95% confidence interval. Black dots denote raw cohort data.

**Figure 4** shows the modelled risk of patients reporting moderate/severe MDADI physical scores (the most highly reported summary score) ≥1 year post-treatment, with cohort results superimposed. For every 1 Gy increase in delivered dose, the absolute risk of moderate/severe symptom reporting increased by 1.5%. Based on this model, we estimate that if doses were corrected back to planned values, absolute risk of self-reported dysphagia symptoms would decrease by ≥5% in 1.2% of patients. Given that the average absolute risk of self-reported dysphagia is 34.9% (SD = 9.3%), dose corrections may decrease relative risk by ≥5% in 23.3% of patients, ≥10% in 3.5% of patients, and ≥15% in 1.2% of patients. The model fit to MDADI composite scores is comparable, indicating a 1.6% decrease in absolute risk per Gy dose correction.

## 4 Discussion

In this study, the strong relationship between delivered pharyngeal constrictor dose and patient-reported dysphagia is comparable to planned dose-PRO associations in the literature (37), yet further indicates that ART dose corrections may be beneficial for reducing dysphagia symptoms. In particular, our logistic regression models suggest that ART corrections may decrease the relative risk of patient-reported physical dysphagia symptoms by ≥5% in 23.3% of patients. We consider these estimates to be conservative. By using doses recalculated on the fraction of last CBCT acquisition to estimate total delivered dose, we make the assumption that patient anatomy was consistent with the last CBCT for all fractions; given that systematic changes in patient and tumor anatomy increase with progression through treatment, our calculations provide an upper bound on estimated inter-fractional dose increases. As corresponding increases in toxicity risk are the reciprocal of dose – calculated by dividing by estimated total delivered dose (e.g., probability of a side effect per Gy) – we obtain a conservative, lower estimate for ART-related toxicity reduction. Therefore, in practice, the toxicity-benefit of ART is likely to be greater than that indicated by our results. To demonstrate this, we performed an additional calculation under the assumption that accumulated delivered dose increases are half that estimated by using the last-acquired CBCT (e.g., assuming systematic anatomical changes increase linearly with time): we found that the absolute risk of moderate/severe MDADI physical scores increased by 1.6% per Gy (*vs*. 1.5% per Gy), with 2.3% (*vs*. 1.2%) of patients having a ≥5% absolute decrease in the risk of self-reported dysphagia and 31.4% (*vs*. 23.3%) of patients having a ≥5% relative decrease in risk.

Xerostomia-reduction is a primary focus of head and neck toxicity studies (2, 5, 38–41); however, dysphagia remains a significant toxicity concern affecting oral intake and health-related quality of life more adversely than xerostomia (42–44). Dysphagia may result in nutritional deficiencies, weight loss, and feeding tube dependence as well as aspiration causing pneumonia and chronic bronchial inflammation (45). When safe to do so, higher prioritization of the pharyngeal constrictor may further reduce dysphagia symptoms (46). For cases where the pharyngeal constrictor is in close proximity to high dose volumes, as was common for our cohort, ART dose corrections may play an important role in dysphagia reduction.

To select patients for ART pharyngeal constrictor dose corrections, our previous work indicates the importance of pre-treatment information, such as planned OAR doses and CTV volumes, and derives clinical guidelines from machine learning modeling (23). Pre-treatment patient selection may streamline ART workflows by allowing patients to be pre-booked for re-CTs and replanning, as compared to interfractional patient monitoring (e.g., assessing weight loss, decrease in face/neck diameter). While many dose-correction strategies exist in the field (47, 48), the work by Hamming-Vrieze et al. cautions against reducing GTV volumes (49), yet OAR doses may be reduced by correcting shifts in steep dose gradients resulting from anatomical changes.

PROs for our cohort are comparable with the existing literature (28, 29) and physician toxicity assessments (1, 2). Our violation formatting is consistent with QUANTEC and other consensus recommendations with respect to dose parameter types and planning objectives, however, future work may consider alternate dose parameter values and OAR such as submandibular and minor salivary glands. Submandibular glands were contoured for our cohort but were prone to deformable image registration errors in our dose estimation workflow, making delivered dose estimates unreliable in these structures (23). The literature indicates that while mean salivary gland dose is strongly associated with saliva flow rates and physician reporting, it is only weakly associated with XQ results ≥12 months post-treatment (38) and may have contributed to the lack of dose-xerostomia associations for our cohort. Although not available for this cohort, OAR sub-contours may further refine dose-PRO associations and ART practices; the literature indicates that the superior pharyngeal constrictors are more strongly associated with late dysphagia (50), with the middle pharyngeal constrictors more strongly associated with acute dysphagia (50) and aspiration (51). Collecting PROs during the course of radiotherapy may build upon known associations between oral cavity dose, mucositis, and quality of life (52, 53).

Limitations of this study include a lack of baseline PRO measures and longitudinal data. We focus on doses to OAR that are most strongly associated with a given toxicity; however, salivary gland dose may further clarify dose-dysphagia associations (54). In estimating the potential benefit of correcting dose violations we make a conservative assumption that OARs may be corrected back to planned values (9). It is possible that corrective gains may be greater in this regard as well (9).

Future work on a larger study cohort may further investigate dose-PRO associations specific to head and neck tumor subsites (e.g., oropharyngeal *vs*. nasopharyngeal disease). We did not observe any statistically significant differences in PRO scores for this cohort with cancer subsite, which may be partially attributed to the similarity of prophylactic nodal volumes among patients of different subsites. As a result, we combined all head and neck cancer subsites into a single analysis; however, subtle differences among subsite groups may exist.

## Data Availability Statement

The datasets presented in this article are not readily available because of the conditions of ethics approval. Requests to access the datasets should be directed to Sarah.Weppler@albertahealthservices.ca.

## Ethics Statement

The studies involving human participants were reviewed and approved by the Health Research Ethics Board of Alberta Cancer Committee. The patients/participants provided their written informed consent to participate in this study.

## Author Contributions

Overall study design was jointly proposed by SW, WS, CS, and HQ. SW and HQ coordinated data collection, including ethics approvals and survey circulation, assisted by AY and NH. SW performed data clustering, statistical analyses, and linear regression model development. LB provided guidance on the analysis of patient-reported outcomes data. SW prepared the initial manuscript with WS, CS, HQ, and LB. All authors contributed to the article and approved the submitted version.

## Funding

This work was supported in part by the Natural Sciences and Engineering Research Council of Canada – Canada Graduate Scholarship (CGS-D) to SW, and the Calgary Foundation - Cadmus Fund.

## Conflict of Interest

The authors declare that the research was conducted in the absence of any commercial or financial relationships that could be construed as a potential conflict of interest.

## Publisher’s Note

All claims expressed in this article are solely those of the authors and do not necessarily represent those of their affiliated organizations, or those of the publisher, the editors and the reviewers. Any product that may be evaluated in this article, or claim that may be made by its manufacturer, is not guaranteed or endorsed by the publisher.
