# Supplementary material for: Patient-Reported Outcomes-Guided Adaptive Radiation Therapy for Head and Neck Cancer
Source: Front Oncol. 2021 Oct 19;11:759724. doi: 10.3389/fonc.2021.759724 (PMC8560706; doi:10.3389/fonc.2021.759724)
Supplement: Supplementary file 1 [file DataSheet_1.docx]

Supplementary Material


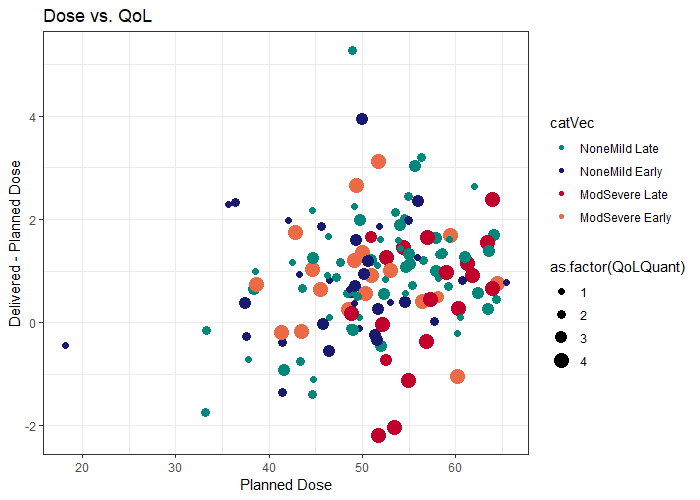


Planned Pharyngeal Constrictor Dmean (Gy)

Delivered – Planned Pharyngeal Constrictor Dmean (Gy)


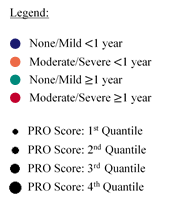


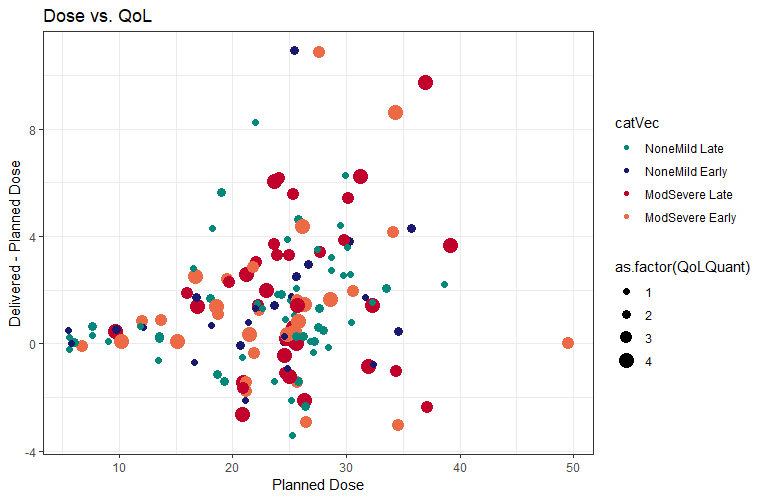


Planned Parotid Gland Dmean (Gy)

Delivered – Planned Parotid Gland Dmean (Gy)

Figure S.1: Differences in planned vs. delivered dose with associated PRO scores. Upper: Pharyngeal constrictor Dmean and MDADI Composite scores. Lower: Parotid gland Dmean and MDASI-HN Dry Mouth scores.
